# Assessing technologies in dementia care: A conceptual health-economic model

**DOI:** 10.1177/13872877251415203

**Published:** 2026-01-28

**Authors:** Jinjing Fu, Ron Handels, Matthieu Arendse, Teis Arets, Ellis Bartholomeus, Marco Blom, Sascha Bolt, Tibor Bosse, Roel Boumans, Debby Gerritsen, Hans Arnold, Wijnand IJsselsteijn, Anne Kolmans, Henk Herman Nap, Baran Polat, Paul Raingeard de la Blétière, Rebecca S. Schaefer, Dirk Steijger, Sander Osstyn, Marjolein de Vugt, Erik Buskens

**Affiliations:** 1 University of Groningen, Department of Epidemiology, University Medical Center Groningen, Groningen, The Netherlands; 2 Alzheimer Centre Limburg, Faculty of Health Medicine and Life Sciences, Mental Health and Neuroscience Research Institute, Department of Psychiatry and Neuropsychology, Maastricht University, Maastricht, The Netherlands; 3 Division of Neurogeriatrics, Department of Neurobiology, Care Sciences and Society, Karolinska Institutet, Solna, Sweden; 4 TanteLouise, Bergen op Zoom, AB, The Netherlands; 5 Department of Industrial Engineering & Innovation Sciences, Eindhoven University of Technology, Eindhoven, The Netherlands; 6 Alzheimer Nederland, Amersfoort, The Netherlands; 7 Tilburg School of Social and Behavioral Sciences, Tilburg University, Tilburg, The Netherlands; 8 Behavioural Science Institute, Radboud University, Nijmegen, The Netherlands; 9 Department of Primary and Community Care, Radboudumc Alzheimer Center, Research Institute for Medical Innovation, Radboud University Medical Center, Nijmegen, The Netherlands; 10 TIGNL BV, Technology Investment Group/JAIN Foundation, Gorinchem, The Netherlands; 11 Department of Primary and Community Care, Radboudumc, Nijmegen, The Netherlands; 12 National Center of Expertise for Long-term Care, Vilans, Utrecht, The Netherlands; 13 Department of Industrial Design Engineering, Delft University of Technology, Delft, The Netherlands; 14 Institute of Psychology, Health, Medical, & Neuropsychology Unit, Leiden University, Leiden, The Netherlands; 15 Academy for Creative and Performing Arts, Leiden University, Leiden, The Netherlands

**Keywords:** Alzheimer's disease, cost-effectiveness, dementia, health-economic model, non-pharmacological interventions, psychosocial interventions, quality of life

## Abstract

**Background:**

Technologies such as assistive devices and social robots show promise in supporting people with dementia and their caregivers. However, their long-term cost-effectiveness remains unclear, and existing health-economic models are limited in capturing the relevant outcomes.

**Objective:**

This study aims to conceptualize a health-economic model to assess the potential impact of care technologies in dementia care on lifetime quality of life and care use.

**Methods:**

We summarized an impact pathway of three care technologies and conceptualized a health-economic model to estimate the long-term impact on quality of life and care use, drawing on literature and multidisciplinary expert input.

**Results:**

We conceptualized a cohort-based Markov state-transition model simulating states of dementia severity progression (mild, moderate, severe), care setting transitions (no formal care, home care, nursing home), and mortality. Intervention effects are modeled through surrogate outcomes such as functional status and caregiver burden associated to care transitions and quality of life.

**Conclusions:**

This model offers a framework for early health technology assessment of assistive technologies in dementia, supporting extrapolation of effects beyond limited trial data. Future work should focus on developing and operationalizing this model, applying it to establish the value of dementia care technologies.

## Introduction

Dementia is a progressive neurodegenerative condition characterized by cognitive decline, behavioral (neuropsychiatric) symptoms, and functional impairment.^1^ This affects the lives of people living with dementia, their caregivers, and the health and social care systems. Currently, 57.4 million people are living with dementia worldwide; this number is estimated to nearly triple to 152.8 million by 2050.^2^ The total societal cost of dementia was estimated to be $1313.4 billion in 2019, half of which was attributable to unpaid informal carers, mainly provided by family.^3^ As population aging progresses and the healthcare labor force cannot keep pace, a shortage is forecast or already imminent.^4^ Addressing the growing impact of dementia requires coordinated efforts to enhance care, support caregivers, and develop sustainable health and social care policies.

A variety of technologies have been developed to support people living with dementia and their caregivers, including assistive devices supporting self-care in daily living, safety monitoring and risk alarm tools, and social robots enhancing emotional well-being.^5–7^ While many of these technological solutions address specific psychosocial or functional needs for their users, dementia care is complex, with challenges and needs constantly varying across individuals and over time.^8^ Rapid advancements in artificial intelligence (AI) can potentially evolve these technologies.^9–12^ By leveraging the learning and predictive power of AI, these technologies can better adapt to the changing and heterogeneous needs of people living with dementia and their caregivers in a precision approach, which may improve quality of life while alleviating caregiver burden.^13–16^

However, limited funding and implementation have long been barriers to accessing promising technologies, restricting their potential impact on dementia care.^17^ In publicly funded systems, adoption typically requires sufficient evidence of cost-effectiveness to demonstrate an intervention's value for money.^18^ Health economic evaluation provides such evidence to inform evidence-based decision-making.^19,20^ Yet, existing health-economic models were predominantly developed for pharmacological interventions and focus on disease progression and modification.^21–23^ These models rarely capture outcomes central to care-support technologies, such as reduction of caregiver burden or improvements in autonomy and well-being.^24–27^ This limitation restricts the assessment and implementation of such technologies^22,24^ and addresses the need for a health-economic models tailed to the evaluation of dementia care solutions.

This study aims to conceptualize a health-economic model to assess the potential impact of care technologies in dementia care on lifetime quality of life and care use. We expect this model to be generalizable to broader care-related innovations and help identify plausible scenarios in which these interventions could be cost-effective to support product development and implementation.

## Methods

Current Heath Technology Assessment (HTA) frameworks offer limited guidance on model conceptualization for non-pharmacological interventions. Therefore, we relied on general recommendations for model conceptualization^28,29^ and model development,^30^ alongside targeted recommendations for economic evaluation of digital health technologies ^31^and artificial intelligence.^32^ An iterative approach was used to establish and refine the conceptual model, drawing on various sources, including our own experience in health economic modeling (RH, EB), insights from systematic reviews of economic modeling related to dementia,^21–23^ relevant literature, and 10 unstructured group discussions with 18 experts in e-health, AI, industrial design, cognitive neuropsychology, virtual agents and social robots, and dementia care in the Netherlands (details provided in Supplemental Material 1). This process for the model conceptualization was summarized into three stages.

### Defining the decision problem and scope

The first step included specifying the technology's intended purpose and target population, the current care pathway (or system pathway), the proposed impact pathway of using the technology, and the expected health, cost, and resource impacts compared to current practice.^31^

This work was carried out within the Dutch national QoLEAD consortium (Quality of Life by Use of Enabling AI in Dementia), with the aim of developing a general framework to assess the health-economic impact of various AI-based technologies. To specify the scope, three AI-based technologies currently under development in QoLEAD were used as the starting cases. Cost-effectiveness analysis (CEA) was used to assess the potential economic and health impacts. The expected health impact was expressed in quality-adjusted life years (QALYs), which relies on a standardized utility measure combined with length of life.^30^ In an iterative approach, we developed the impact pathways of the three technologies in a diagram to visualize how they affected cost (resource use) and QALY over time. This diagram was refined using insights from the Andersen Behavioral Model^33^ and expert input. The Andersen Model, a widely used framework for explaining health service utilization, helped explain the proposed pathway between the technologies and care use.^33–36^ Experts were asked to assess whether the diagrams sufficiently captured the intended impact pathways of their respective technologies and to contribute suggestions for improvement.

### Structuring a decision model

The impact pathway formed the basis of the model structure. We sought to balance the representation of the real-world complexity, but with sufficient simplicity to match available data.^29^ The Markov state-transition model, based on dementia severity stages and institutionalization, like the IPECAD model approach,^37^ was used as the foundation of the conceptual model for its transparency and open-source nature. Recent systematic reviews of model-based economic evaluations of dementia interventions^21–23^^,38^ provided insights into existing modeling approaches and highlighted areas for improvement, specifically for non-pharmacological interventions.^21^ We considered this improvement recommendations related to our specific decision-making context. Experts assessed if the model structure appropriately represented the dementia care pathway in the Netherlands and was relevant to the technology's intended purpose.

### Evidence synthesis and extrapolation strategy

Since this was a conceptualization stage, evidence generation was not yet conducted. However, a critical step in model development is identifying and synthesizing evidence to ensure that the model can be operationalized.^39^ To assess feasibility, an ad-hoc literature search and expert knowledge were gathered to identify longitudinal data and empirical evidence aligning with the model structure and study scope. Effectiveness outcomes that can reasonably be obtained from short-term pilot studies were selected by experts. An extrapolation strategy was developed to synthesize effectiveness evidence and extrapolate it into lifetime costs and QALY, illustrating how the model captures the impact of the technology by integrating potential observational evidence, expert opinion, and empirical evidence.

## Results

We present the conceptual model by outlining its key features according to the three conceptualization stages outlined in the Methods. As the model conceptualization was directly shaped by the scope of the exemplar interventions, we begin by describing the dementia care context and the specific technologies under development. These interventions informed the development of a generalized impact pathway, which in turn guided structural model choices and extrapolation strategy.

### The decision problem and scope

#### Description of dementia progression and current care pathway

The slow, progressive nature of dementia leads to a gradual increased need for social care.

#### Care support over time

Care for people with dementia typically begins with support from informal caregivers, mostly family members.^40^ As the disease advances and the person's care needs exceed the informal caregiver's capacity, formal care support, such as home nursing support, domestic assistance, and day care, becomes necessary in addition to informal care. Eventually, when dementia symptoms become severe, admission to a 24-h institutional care facility, such as a nursing home, is needed as care exceeds what can be managed within the community.^41^ These informal and formal care resource use represent key cost drivers in dementia care.^3^ The transitions between these care settings are associated with significant changes in resource use and quality of life, with studies showing a progressive decline in quality of life and a steep cost rise in institutional care settings.^42,43^ The organization and timing of these care transitions vary across healthcare systems. The three selected care types, no formal care, home care, and institutional care, have been used in previous research to describe care transitions in the Netherlands.^44,45^

#### Scope: intended purpose and target population of AI-based technologies

Three AI-based technologies are described below.

- An interactive social agent to support activities of daily living with a focus on cooking tasks. It uses an assistance system to provide tailored cooking instructions, recipe suggestions, and safety monitoring for users. The social agent uses an AI-based speech recognition system to recognize dementia severity stages and adapt its assistance. It aims to maintain the independence of people living with dementia and enhance their autonomy. It also aims to reduce the caregiver's burden and the need for formal care support (e.g., meal service and domestic assistance).
- A musical social robot guides reminiscence and enriches social activities based on an AI algorithm, delivering personalized music experiences and social engagement activities for people living with dementia. It aims to simulate cognitive function and improve emotions and quality of life.
- An AI system analyzes nursing home clinical records to predict unexplained behavior or a drop in quality-of-life risk and provide early alerts and tailored recommendations to care staff.

The first two technologies aim to support individuals with mild to moderate dementia primarily in community settings, whereas the third technology is not restricted to a particular care setting or dementia severity*.*

#### Impact pathway from AI-based technologies to health-economic outcomes

**Figure 1.**
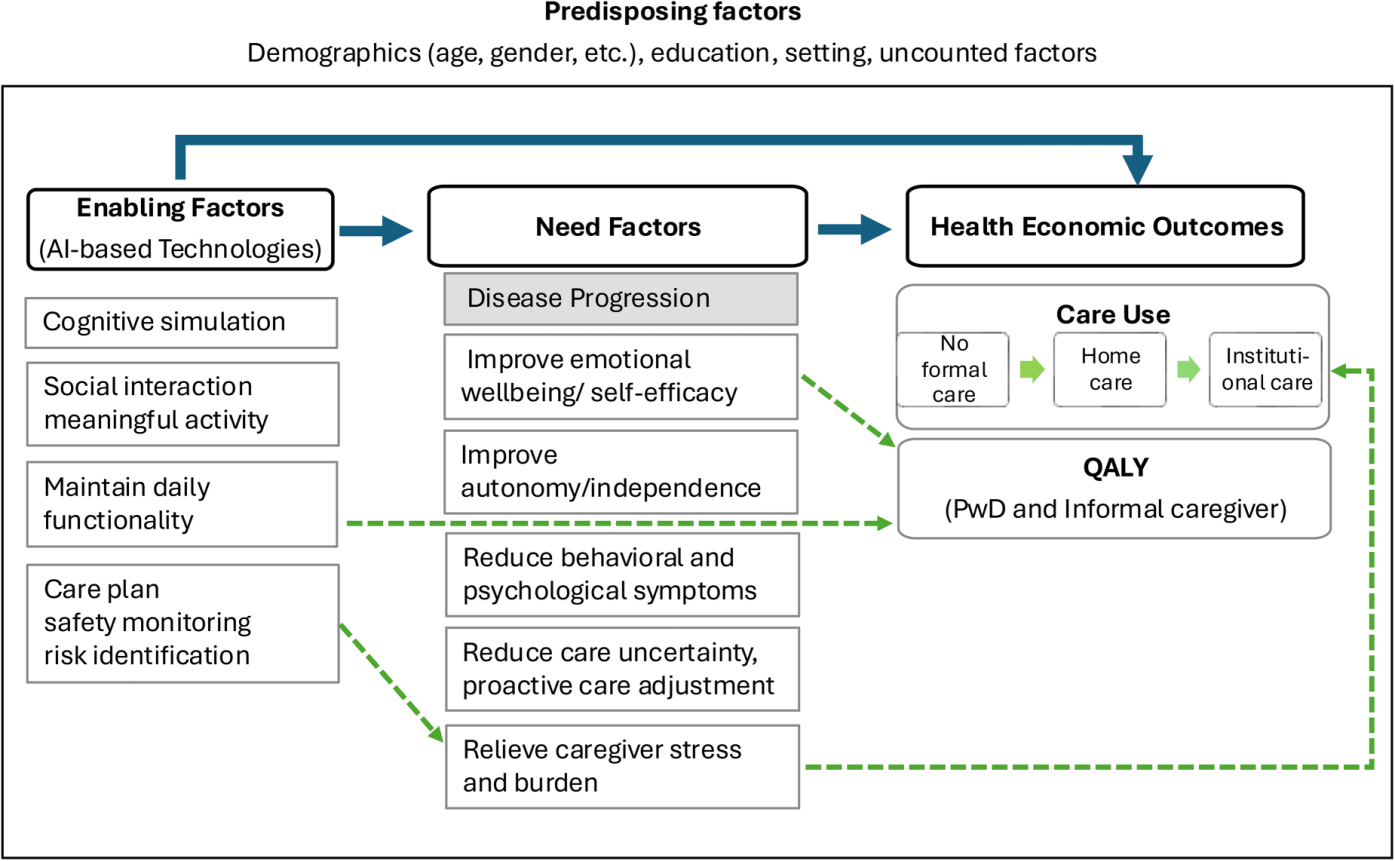
Impact Pathway from AI-based technologies to health-economic outcomes. Solid arrows represent the generalized impact pathway, showing how AI-based technologies act as enabling factors by influencing need factors and affecting care use and quality-adjusted life years (QALY) outcomes for both people with dementia and their caregivers. Transitions between care settings (from no formal care to home care and institutional care), representing care use in The Netherlands. Dashed arrows highlight the specific interactions of factors along the impact pathway of a social agent, which supports individuals in maintaining daily functionality, thereby influencing multiple need factors and delaying transitions to more intensive care settings. It may directly improve the quality of life. Predisposing factors (e.g., demographics, education, setting) exert an overarching influence across enabling, need, and outcome factors. Unless otherwise specified, “caregiver” refers to both informal and professional caregivers. QALY, quality-adjusted life years.

Figure 1 summarizes the generalized impact pathway illustrating how the three technologies lead to an impact on health economic outcomes. Although the three technologies differ in their specific functions, they offer four key areas of support: maintaining daily functionality (A), enriching social interaction and meaningful activities (A, B), providing safety monitoring and risk identification (A, C), and stimulating cognitive function (B). Through these areas of support, all three technologies aim to address the psychosocial and functional needs of people living with dementia or their caregivers, ultimately improving overall quality of life for both.

The relationship between technologies and care use was structured using the Anderson behavioral model, which explains health resource utilization by 3 key factors, including predisposing, need, and enabling factors.^33–36^ Predisposing factors relate to demographic, social and mental factors (e.g., age, education and attitudes); need factors relate to health status, functional state and illness symptoms (e.g., cognitive decline and mobility limitations) that drive professionally evaluated medical or care need, and also refer to the perceived illness or burden experienced by the individual or caregiver, which may influence care-seeking behavior; enabling factors relate to financial and organizational conditions that enable service utilization (e.g., insurance coverage that affects frequency of in-home support service use, hospital or service provider density that affects waiting time of admission and access to telehealth monitoring).^33–36^ We identified the technologies as enabling factors that may either directly affect the use of care or indirectly, by affecting the need factors, which leads to downstream effects on care use.

Along this generalized impact pathway, the interactions between specific factors can vary depending on the purpose and function of the technology. We use the social agent as an example to illustrate these interactions (as indicated with blue dashed lines in Figure 1). This agent provides guidance and safety monitoring to support people living with dementia with meal preparation. By supporting them to maintain daily functionality, the technology could, in the short term, preserve autonomy. This, in turn, reduced caregiving hours required from informal caregivers and alleviated their mental strain on safety and/or reduced formal care support (e.g., domestic assistance, meal delivery). Furthermore, by reducing informal caregiver burden, the technology may increase their perseverance time, thereby in the long-term helping to sustain care in the community, potentially postponing nursing home placement. (Examples of the other two technologies are included in Supplemental Material 1).

While specific interactions vary, technology developers consistently viewed it as unlikely that these interventions would affect the need factor, the biological progression of dementia. For example, although the music robot includes reminiscence features intended to stimulate cognition, these are primarily designed to enhance mood, engagement, or quality of life, rather than to meaningfully alter the course of disease progression. However, such technologies may still affect illness-related need factors (such as maintaining functional ability or reducing caregiver burden) and influence how long a person living with dementia can remain in a lower-intensity care setting before transitioning to intensive (institutional) services.^8,13,14^ Given the above considerations, we found it key for the model to reflect how the AI technologies would change transitions across care types.^45^

### Structuring the decision model

#### Model structure

Guided by the impact pathway, the model structure for care technologies centers around the care pathway, rather than the biological or symptomatic progression of dementia. However, modeling dementia progression remains important because the likelihood of formal and institutional care use increases as dementia deteriorates.^44,46^ Moreover, technologies are often designed for use at a specific disease severity. For example, technology acceptance was deemed to be improved when exposed to it already in an earlier dementia stage. Therefore, disease progression was reflected in the model structure to allow care transitions to be conditional on disease severity. Disease progression was reflected as mild, moderate, or severe dementia, similar to the often-used model structure, such as the IPECAD model.^47^

**Figure 2.**
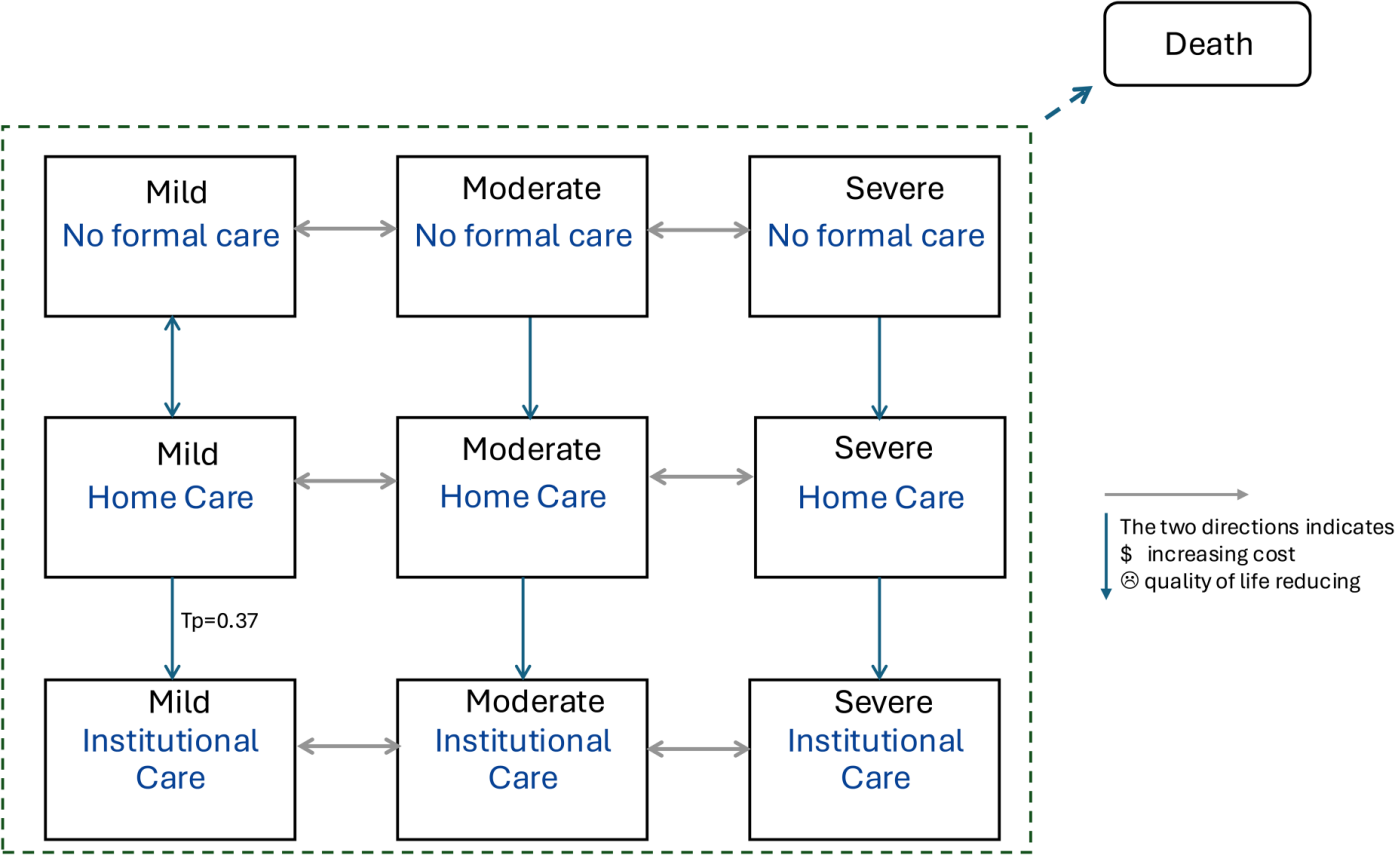
Visual representation of the Markov model structure reflecting 9 combination states of disease severity and care setting, and 1 state of death. Each state is associated with its state value: ☹: hypothesized utility-based quality of life $: hypothesized care costs.

A Markov state-transition model is selected to simulate dementia progression represented by transitions between mild, moderate, and severe severity states, and to simulate the care pathway by transitions between care states: no formal care, home care, and institutional care (with definition provided in Supplemental Material 3). Each two states contain a transition probability, which reflects the probability of moving from one state to the other within a given period. The transition probability between care states is conditional on dementia severity. From any state, there is also a risk of death, which is conditional on both dementia severity and the type of care received. (See Figure 2 for a representation of the model structure and key transitions.) Each state has a corresponding mean utility-based quality of life for the person with dementia and their caregivers, along with mean care costs over a given period. A Markov model runs cycle by cycle, applying the transition probabilities to estimate the time individuals spent in each state and their corresponding cumulative QALYs and care costs.

→ indicates the transition direction. For simplicity, we omitted transitions skipping disease states (e.g., mild to severe), we assumed transitions between care settings are unidirectional (i.e., only towards a higher intensive care setting) in the moderate and severe stage, and we omitted transitions to disease state and care setting simultaneously (i.e., “diagonal” transitions). For illustration, the transition probability between mild home care and mild institutional care in one year is marked as Tp = 0.37. In the full model, each pair of states is connected by a transition probability, representing the likelihood of moving from one state to another within a given period.

#### Choice of model type

Considering the features of AI, a micro-simulation model type was also considered appropriate in this decision context. The strength of a micro-simulation model is in its ability to reflect heterogeneity at the individual level and provide more transparency compared to a Markov model with many states or attributes. Notably, AI-based technologies address individual needs, whereas a state-transition Markov model typically tracks cohort average outcomes. Despite this, we consider the Markov model appropriate for the assessment because this study focuses on assessing the average impact of the intervention for the target population, rather than the variability in how the intervention addresses different needs. For instance, while an AI-based cooking agent may offer individualized support, such as a reminder for the next step in the cooking process for one person or automatic stove shutdown for the other, the outcome of interest will be the overall effect of the intervention in its target population. Assessments of heterogeneity, such as outcome differences by sex or age, can be evaluated by running the model with subgroup-specific inputs and weighting the results. Given the early stage of technology development and limited evidence to provide meaningful modeling inputs, a Markov model was again deemed more suitable.

### Evidence synthesis and extrapolation strategy

#### Implementation of intervention effectiveness estimates

The model begins with a baseline set of transition probabilities, state-specific values applied to both the usual care strategy (i.e., control group) and the intervention strategy. The impact of the technology is reflected by its effect on (1) state-specific values (e.g., the music robot may improve utility-based quality of life/reduce care hours in the state of mild dementia with no formal care in the intervention strategy) and/or (2) transition probabilities between states (e.g., the social interactive agent may lower the risk of initiating home care) in the intervention strategy.

#### Extrapolation of short-term effectiveness evidence over a lifetime horizon

However, limited evidence will be available on quality of life and care transitions as these are typically expected during long-term exposure or beyond. Reviews of effectiveness evaluation of digital health technology, smart home, and assistive technology trials showed that short-term studies (e.g., 6 weeks to 6 months) often focus on end-of-intervention qualitative evaluations, assessing user satisfaction and acceptability.^8,15,48^ Quantitative measures in these studies include quality of life, depression, carer burden, resource use (e.g., caregiving time), functional abilities, and behavioral changes such as agitation and aggression.^15,23,48^ In longer-term studies, with over 24 months of follow-up, time to nursing home admission was frequently used to measure impact.^15,23,49–51^

In this study context, the effectiveness of technologies early in their development is expected to be assessed through small-scale, non-randomized pilot studies with relatively short-term (e.g., 2–12 weeks) follow-up on outcomes relatively close to the targeted effect domain of the intervention (expected outcomes are listed in Supplemental Table 2). Short-term effect estimates from the pilot study may be extrapolated by the model to long-term effects in two approaches (also listed in Supplemental Table 2).

- Direct application: Pilot effect estimates for outcomes that directly reflect state-specific values or transition probabilities, such as utility-based quality of life, caregiving hours, resource utilization, and risk of nursing home admission, can be applied to the intervention strategy in the model beyond the pilot's follow-up period. Expert opinion may inform assumptions about how long such effects persist.
- Surrogate translation: Pilot effect estimates for outcomes that do not directly reflect state-specific values or transition probabilities; these estimates may act as surrogate outcomes and still be mapped onto model parameters using evidence from the literature. For example, a pilot effect estimate on reducing caregiver burden could be translated to a lower risk of nursing home placement (typically a long-term outcome) using the relative risk of nursing home placement related to caregiver burden observed from the literature.^52^ Similarly, changes in behavioral symptoms can be linked to changes in utility-based quality of life.^53^ As with the previous approach, assumptions are needed regarding how long these effects persist beyond the pilot period.

#### Surrogates outcomes

The selection of surrogates and the empirical evidence to support extrapolation are crucial to ensure valid long-term prediction. While existing recommendations on the use of surrogate outcomes in HTA^54^ primarily focus on biomarkers and biological surrogacy, this recommendation was adapted to apply to non-pharmacological interventions by drawing insights from NICE recommendations for digital health technologies.^31^ We followed three steps to identify potential surrogate outcomes.

First, candidate surrogates were selected by experts considering whether the technology plausibly affects the surrogate, and whether that surrogate is associated with a state-specific value (utility-based quality of life, care use) or a transition probability (Supplemental Table 2), and the surrogacy relationships were justified through the technology impact pathway. Second, the surrogate must hold meaningful value for key stakeholders, including people living with dementia and their caregivers, to support decision-making on the reimbursement/acceptance of a technology. Harding et al. provided a core outcome set for non-pharmacological interventions for people living with dementia at home, and it was used as a comprehensive list to consider surrogates from.^25^ Third, existing literature informed on the association between surrogate and state-specific value or transition (e.g., nursing home admission).^36,41,55–58^

The potential surrogate outcomes for care transitions include caregiver burden and coping abilities, the ability to perform activities of daily living, and behavioral and psychological symptoms.^44,59,60^ Kerpershoek et al. explored the ACTIFCARE cohort and identified significant predictors of formal care access, including disease severity, hours spent on informal care, adjusted for other factors.^59^ Coley et al. identified predictors of accessing home care, including cognition, behavioral and psychological symptoms, and caregivers’ burden in the ICTUS cohort, adjusted for other factors.^44^ Kraijo et al. and Richters et al. explored caregiver perseverance time, which reflects perceived caregiver burden and coping ability as a potential predictor of nursing home admission, adjusted for other factors.^60,61^

Depending on available evidence, surrogate effects could be applied in two ways: as risk ratio modifying transition rates between care settings, or as absolute change that directly adjusts care hours or utility values to reflect cost and quality-of-life impacts. It must be applied cautiously to avoid double-counting their effects. For example, the ability to perform daily activities measured by ADCS-ADL (Alzheimer's Disease Cooperative Study - Activities of Daily Living) is associated with utility-based quality of life measures (a point increase in ADCS-ADL corresponding to 0.008 utility gain),^62,63^ while it is also associated with the risk of nursing home placement.^52^ If quality of life is directly measured (e.g., via EQ-5D), using iADL to estimate utility changes would duplicate the effect. Even without direct QoL measurement, using iADL to predict both utility and transitions risks conflates causal pathways (e.g., QoL decline from functional loss versus QoL changes from delayed institutionalization).

Functional ability and behavioral symptom measures could also be used to estimate treatment effects and disease progression in a multi-domain model like IPECAD.^47^ However, this approach is problematic in our context. This study assumes that interventions like the described technologies are effective only during active use. Once discontinued, their effects are assumed no longer sustained, and individuals in the intervention group revert to the same state as those who have never received the intervention. For example, a technology assisting people living with dementia to take medication and prepare daily meals independently may improve the instrumental activities of daily living (iADL) score that reflects functional ability. In a multi-domain model, changes in iADL are simulated as delayed disease progression, leading to reduced costs associated with function-related care (such as informal support). However, if the intervention's effectiveness wanes, a Markov model would still reflect the previously gained benefits. Additionally, modeling functionality in the dementia progression path could lead to indirect mortality benefits. Based on these considerations, functional and behavioral measures were used to predict the intervention effects on care transitions instead of on disease progression.

## Discussion

Drawing from expert input and literature, we developed a generalized impact pathway that integrates three conceptually and functionally distinct care technologies. Despite their different intended purpose, these technologies influence care use and quality of life through shared mechanisms, such as improving independence in daily living and reducing caregiver burden. This pathway was not only provided a foundation for understanding how intervention effects may unfold over time and directly informed the structure of the model.

We conceptualized a Markov-type state transition model, with disease progression reflected by transitions between mild, moderate, and severe dementia states, with care setting reflected by transitions between no formal care, home care, and institutional care, alongside mortality risk from each state. Each state is associated with care costs and utility-based quality of life values. The model enables the simulation of long-term (extrapolated) intervention effects through surrogate outcomes to predict changes in the probability of transition between care states or sustained effects on state values. The impact pathway plays a critical role in justifying this surrogate-based approach. It also offers a transferable framework for evaluating broader carer-related innovations, such as case management and informal caregiver support programs that follow similar impact pathway. However, its relevance to lifestyle, screening, or diagnostic interventions may be limited. Empirical implementation and pilot simulation are essential steps for future research to demonstrate the model's feasibility and performance.

Our approach differs from existing frameworks in several aspects. Compared to the IPECAD multi-domain modeling and the model developed by Jutkowitz et al.,^47,64^ both of which offer a comprehensive description of dementia progression, incorporating cognitive, functional, and behavioral domains, we simplified the description of dementia progression by focusing solely on the cognitive domain. This choice reflects current lack of country-specific data in care transitions conditional on symptoms across multiple domains. Structurally, our model resembled Neumann's model^65^ and many similar successors, but we added an additional care setting state (no formal care) to align with care pathways in the Netherlands. While previous studies^49,50,64^ have evaluated community-based, non-pharmacological support services by simulating intervention effects through institutionalization changes, they relied on long-term effectiveness evidence on care usage (admission to nursing home) from the trial. Our model offers a theoretical structure that uses surrogate outcomes to estimate these long-term impacts when such evidence is lacking in early technology development. Unlike both IPECAD^47^ and the Alzheimer's Disease Archimedes Condition-Event (AD-ACE) simulator,^66^ which model treatment effects through disease progression, our model emphasizes care pathways, aligning with most non-pharmacological interventions’ focus on addressing psychosocial and functional needs of people living with dementia and their caregivers rather than disease symptoms alone.

This modeling approach reflects assumptions about intervention effects in this study context. While technology experts suggested that interventions may simulate cognitive function through increased activity and social engagement, we assumed these improvements would be insufficient to alter disease progression in the model. The approach is aligned with the NICE approach in health technology assessment of non-pharmacological interventions such as cognitive stimulation therapy.^62^ It acknowledges potential cognitive benefits on quality of life while maintaining conservative assumptions of not affecting disease progression. However, this assumption should be revisited if future evidence demonstrates an impact on disease progression.

While conceptualizing the model, data availability was considered to describe care transition and effectiveness outcomes, which may seem inconsistent with guidelines that recommend structuring a conceptual model around the decision problem, not data availability.^29,30,67^ However, guidelines also suggested that “structure should reflect the relationship between the inputs (e.g., natural history of diseases, treatment pathway, epidemiological data, effectiveness data) and the outcomes (such as the number of health events, outcomes summary of cost-effectiveness) required by the decision maker”.^29,30,67^ Pragmatic consideration of data availability is crucial for statistically describing these relationships and ensuring the feasibility of modeling.^68^ For example, data availability to describe natural disease progression should be a prior consideration in model structure, as without an empirical basis, constructing a model is likely not feasible. This is regarded as a prior consideration necessary for model development rather than general structural considerations like time horizon or model cycle length.^39,68^ Based on this consideration, datasets and empirical models describing risk factors and care transitions were identified to inform the selection of relevant states, events, and risk factors for inclusion. Additionally, empirical evidence was assessed to generate evidence linking surrogate outcomes to long-term care transitions. Without these details, simulating the mechanistic components of the model would not be feasible.

Although the current model was developed in the Dutch context, it was designed to be adaptable to other health systems in high-income countries especially in the European Union. However, care states need to be clearly defined by the care trajectory in different health systems. Adaptation may involve adjusting parameters such as transition probabilities, utility values, resource use, the distribution of population in different care setting and severity states. Providing full cross-country applications was beyond the scope of this study; however, such analyses represent a key direction for future research.

AI-specific components were considered during the conceptualization process based on the recent CHEERS checklist for Interventions that use AI^32^ and a broad health technology assessment framework of medical AI.^69^ Given that these technologies have not been fully developed, AI-specific features such as continuous learning were not incorporated into the current model. However, they are recommended to be examined by the guidelines.

It is widely recognized that enabling individuals with dementia to remain in the community not only reduces the economic burden on the healthcare system but also enhances their quality of life, which is why many non-pharmacological interventions are designed to support community-based care.^70^ Our conceptual model is based on this rationale. However, this may not hold universally, given the heterogeneity of people living with dementia and their care situation in the community. Furthermore, measuring the subjective well-being of people living with dementia, especially in later stages, is challenging and remains a debated concept.^71^ Future research should explore these assumptions and consider the variability in institutional care quality and individual experiences. Until further evidence is obtained, the sensitivity of health-economic outcomes to these assumptions should be assessed.

### Limitations

Our study has several limitations. First, we held unstructured focus group interviews with a limited number of experts rather than more comprehensive methods such as Delphi. Additionally, for the patient and public perspective, we relied on literature rather than focus groups specific to our study purpose and context.^25^ These choices may have introduced bias by limiting the diversity and contextual relevance of perspectives captured. We expect that any bias introduced is more likely to have led to missing some relevant outcomes rather than systematically favoring particular outcomes. However, future research may consider involving patients and caregivers directly through Delphi or structured focus group approaches to enhance the generalizability and user-centeredness of the framework.

Second, the use of surrogate outcomes to predict care transitions introduces uncertainty, as it relies on the assumption that associations are fully causal. The observational nature of the pilot, in contrast to randomized designs, provides only limited support for causality, as there remains uncertainty whether an effect is adjusted for all possible confounders. Findings from several systematic reviews and studies on factors associated with institutionalization in people with dementia show some inconsistency,^36,41,52,55,72^ highlighting the challenge of establishing robust predictive relationships. Sufficient evidence on predictive factors that are specifically applicable to the Dutch context (risk of institutional care admission) remains scarce, which further limits the strength of the estimation. Additionally, some evidence in our model is based on studies involving individuals with informal caregiver support,^25,26,47,48^ limiting the model's applicability to people living with dementia who lack informal caregiver support. Evidence is likely biased towards low-burdened informal caregivers who have no limitation in participation. Future empirical implementation should include extensive sensitivity analysis to assess the robustness of surrogate outcome assumptions and strengthen casual inference regarding their long-term predictive validity. Specifically, scenario analyses (e.g., best case and worst case) based on published literature and expert opinion can examine how changes in surrogate validity may influence cost-effectiveness conclusions and policy implications. If such conclusions are highly sensitive to uncertainty around the surrogate outcome it stresses the importance of future research to collected (randomized) longitudinal evidence for validating surrogate outcomes.^54,73^

Third, there are inherent limitations in using cost-effectiveness analysis, particularly when value is primarily measured through utility-based quality of life outcomes and resource use. However, the value of these technologies extends beyond those measures.^74^ Expert discussions highlighted several potentially important factors that were not included in the current model, either due to a lack of available data, their indirect relationship with modeled outcomes, or conceptual complexity. These include improvements in caregiver experience, reductions in staff burden, and broader considerations such as equity, scientific spillovers, and ethical issues like user autonomy and data privacy. Methodological guidance for implementing equity considerations in health economic evaluations outlines practical approaches, such as integrating equity weights and conducting subgroup analyses within cost-effectiveness frameworks.^75^ However, challenges around data availability may limit the extent to which equity can be rigorously incorporated in practice. Contextual factors, such as effect differences between lab and real-world environments, digital literacy, and the presence or absence of informal care, also remain unaccounted for. We recognize growing interest in approaches like multi-criteria decision analysis (MCDA), particularly in elderly care, where diverse outcomes matter to multiple stakeholders.^76^ While remaining open to the integration of broader value elements through methods like MCDA, we consider this model providing a starting point for value-based assessment of non-pharmacological interventions.

Although this framework adopts a societal perspective and includes informal caregiving hours as part of total cost, informal and indirect costs remain an underrepresented component to address the economic burden of dementia care. Informal care costs have typically been expressed in monetary terms related to time providing support for activity of daily living, while other elements such as supervision and productivity loss have been studied to a much lesser extent and are not captured in the current version of the model. Although not captured in this model, broader fiscal implications such as potential increases in pension expenditures resulting from extended survival and improved care remain an important economic consideration.

It is difficult to judge whether these limitations lead to an over- or underestimation of the impact of a technology. The assumption of full causality and selective evidence from low-burdened informal caregivers may lead to overestimation, while the unstructured focus groups and limited surrogate outcomes may lead to underestimation. Nevertheless, our conceptual model addresses key limitations identified in previous models for non-pharmacological interventions in dementia, contributing to ongoing methodological development in this area.

Although formal HTA is not yet mandated for reimbursement of such technologies, this anticipatory approach aligns with international efforts aiming to systematize reimbursement and funding decisions.^69,77^ There is increasing interest in expanding the role of HTA in the assessment of non-pharmacological interventions and growing recognition of the need for robust evidence early in the innovation process.^69,77^ This study supports the development of new technologies in dementia care that are relevant and more likely to achieve sustainable adoption within care and social systems.

### Conclusion

This model offers a potentially generalizable framework for evaluating the cost-effectiveness of non-pharmacological technologies in dementia care. By focusing on care transitions and incorporating surrogate outcomes, it enables the extrapolation of long-term impact from short-term data, supporting early evaluations even when evidence is limited. Its structure allows for the integration of multiple outcome domains of intervention effects, making it relevant for both technology developers and decision-makers. As such, it can inform design choices, reimbursement considerations, and broader policy discussions on innovation in dementia care.

## Supplemental Material

sj-docx-1-alz-10.1177_13872877251415203 - Supplemental material for Assessing technologies in dementia care: A conceptual health-economic model Supplemental material, sj-docx-1-alz-10.1177_13872877251415203 for Assessing technologies in dementia care: A conceptual health-economic model by Jinjing Fu, Ron Handels, Matthieu Arendse, Teis Arets, Ellis Bartholomeus, Marco Blom, Sascha Bolt, Tibor Bosse, Roel Boumans, Debby Gerritsen, Hans Arnold, Wijnand IJsselsteijn, Anne Kolmans, Henk Herman Nap, Baran Polat, Paul Raingeard de la Blétière, Rebecca S. Schaefer, Dirk Steijger, Sander Osstyn, Marjolein de Vugt and Erik Buskens in Journal of Alzheimer's Disease

## References

[1] Mckhann GM Knopman DS Chertkow H , et al. The diagnosis of dementia due to Alzheimer's disease: recommendations from the National Institute on Aging-Alzheimer's Association workgroups on diagnostic guidelines for Alzheimer's disease. Alzheimers Dement 2011; 7: 263–269. 21514250 10.1016/j.jalz.2011.03.005 PMC3312024

[2] GBD 2019 Dementia Forecasting Collaborators. Estimation of the global prevalence of dementia in 2019 and forecasted prevalence in 2050: an analysis for the Global Burden of Disease Study 2019. Lancet Public Health 2022; 7: e105–e125. 10.1016/S2468-2667(21)00249-8 PMC8810394 34998485

[3] Wimo A Seeher K Cataldi R , et al. The worldwide costs of dementia in 2019. Alzheimers Dement 2023; 19: 2865–2873. 36617519 10.1002/alz.12901 PMC10842637

[4] Zapata T Azzopardi Muscat N Falkenbach M , et al. From great attrition to great attraction: countering the great resignation of health and care workers. Eurohealth (Lond) 2023; 29: 6–10.

[5] Lorenz K Freddolino PP Comas-Herrera A , et al. Technology-based tools and services for people with dementia and carers: mapping technology onto the dementia care pathway. Dementia (London) 2019; 18: 725–741. 28178858 10.1177/1471301217691617

[6] Ipakchian Askari S Vasseur D Hofstede B , et al. Mapping dementia care technology: tailored digital solutions across stages. Int Med Educ 2024; 3: 140–151.

[7] Cheng JY Nurul SBMS Cheng LJ , et al. Effectiveness of technology-delivered psychosocial interventions for family caregivers of patients with dementia: a systematic review, meta-analysis and meta-regression. Int J Ment Health Nurs 2024; 33: 1796–1816. 39034437 10.1111/inm.13390

[8] Meiland F Innes A Mountain G , et al. Technologies to support community-dwelling persons with dementia: a position paper on issues regarding development, usability, effectiveness and cost-effectiveness, deployment, and ethics. JMIR Rehabil Assist Technol 2017; 4: e1. 10.2196/rehab.6376 PMC5454557 28582262

[9] Su Z Bentley BL McDonnell D , et al. 6G And artificial intelligence technologies for dementia care: literature review and practical analysis. J Med Internet Res 2022; 24: e30503. 10.2196/30503 PMC9096635 35475733

[10] Kameyama M Umeda-Kameyama Y . Applications of artificial intelligence in dementia. Geriatr Gerontol Int 2024; 24: 25–30. 10.1111/ggi.14709 PMC11503597 37916614

[11] Qi J Wu C Yang L , et al. Artificial intelligence (AI) for home support interventions in dementia: a scoping review protocol. BMJ Open 2022; 12: e062604. 10.1136/bmjopen-2022-062604 PMC9494582 36130752

[12] Borna S Maniaci MJ Haider CR , et al. Artificial intelligence support for informal patient caregivers: a systematic review. Bioengineering (Basel) 2024; 11: 483. 38790350 10.3390/bioengineering11050483 PMC11118398

[13] de Joode E van Heugten C Verhey F , et al. Efficacy and usability of assistive technology for patients with cognitive deficits: a systematic review. Clin Rehabil 2010; 24: 701–714. 20543021 10.1177/0269215510367551

[14] Ienca M Jotterand F Elger B , et al. Intelligent assistive technology for Alzheimer's disease and other dementias: a systematic review. J Alzheimers Dis 2017; 60: 333. 28869482 10.3233/JAD-179005

[15] Moyle W Murfield J Lion K . The effectiveness of smart home technologies to support the health outcomes of community-dwelling older adults living with dementia: a scoping review. Int J Med Inform 2021; 153: 104513. 34116363 10.1016/j.ijmedinf.2021.104513

[16] Neal I Du Toit SHJ Lovarini M . The use of technology to promote meaningful engagement for adults with dementia in residential aged care: a scoping review. Int Psychogeriatr 2020; 32: 913–935. 31547900 10.1017/S1041610219001388

[17] Boyle LD Husebo BS Vislapuu M . Promotors and barriers to the implementation and adoption of assistive technology and telecare for people with dementia and their caregivers: a systematic review of the literature. BMC Health Serv Res 2022; 22: 1573. 36550456 10.1186/s12913-022-08968-2 PMC9780101

[18] Zorginstituut Nederland. Guideline for economic evaluations in healthcare. Diemen, the Netherlands: Zorginstituut Nederland, 2016.

[19] Kluytmans A Tummers M van der Wilt GJ , et al. Early assessment of proof-of-problem to guide health innovation. Value Health 2019; 22: 601–606. 31104741 10.1016/j.jval.2018.11.011

[20] Grutters JPC Govers T Nijboer J , et al. Problems and promises of health technologies: the role of early health economic modeling. Int J Health Policy Manag 2019; 8: 575–582. 31657184 10.15171/ijhpm.2019.36 PMC6819627

[21] Sopina E Sorensen J . Decision modelling of non-pharmacological interventions for individuals with dementia: a systematic review of methodologies. Health Econ Rev 2018; 8: 8. 29582186 10.1186/s13561-018-0192-8 PMC6755571

[22] Darab MG Engel L Henzler D , et al. Model-based economic evaluations of interventions for dementia: an updated systematic review and quality assessment. Appl Health Econ Health Policy 2024; 22: 503–525. 38554246 10.1007/s40258-024-00878-0 PMC11178626

[23] Eaglestone G Gkaintatzi E Jiang H , et al. Cost-effectiveness of non-pharmacological interventions for mild cognitive impairment and dementia: a systematic review of economic evaluations and a review of reviews. Pharmacoecon Open 2023; 7: 887–914. 37747616 10.1007/s41669-023-00440-z PMC10721583

[24] Boccardi M Handels R Gold M , et al. Clinical research in dementia: a perspective on implementing innovation. Alzheimers Dement 2022; 18: 2352–2367. 35325508 10.1002/alz.12622

[25] Harding AJE Morbey H Ahmed F , et al. A core outcome set for nonpharmacological community-based interventions for people living with dementia at home: a systematic review of outcome measurement instruments. Gerontologist 2021; 61: e435–e448. 10.1093/geront/gnaa071 PMC8599310 32583858

[26] Olazarán J Reisberg B Clare L , et al. Nonpharmacological therapies in Alzheimer's disease: a systematic review of efficacy. Dement Geriatr Cogn 2010; 30: 161–178. 10.1159/000316119 20838046

[27] Knapp M Shehaj X Wong G . Digital interventions for people with dementia and carers: effective, cost-effective and equitable? Neurodegener Dis Manag 2022; 12: 215–219. 35833456 10.2217/nmt-2022-0025 PMC9517957

[28] Caro JJ Briggs AH Siebert U , et al. Modeling good research practices–overview: a report of the ISPOR-SMDM Modeling Good Research Practices Task Force–1. Value Health 2012; 15: 796–803. 22999128 10.1016/j.jval.2012.06.012

[29] Roberts M Russell LB Paltiel AD , et al. Conceptualizing a model: a report of the ISPOR-SMDM Modeling Good Research Practices Task Force-2. Value Health 2012; 15: 804–811. 22999129 10.1016/j.jval.2012.06.016 PMC4207095

[30] Briggs A Claxton K Sculpher M . Decision modelling for health economic evaluation. Oxford, UK: Oxford University Press, 2006.

[31] National Institute for Health and Care Excellence (NICE). Evidence standards framework for digital health technologies. London, UK: NICE, 2022.

[32] Elvidge J Hawksworth C Zemplenyi A , et al. Consolidated health economic evaluation reporting standards for interventions that use artificial intelligence (CHEERS-AI). Value Health 2024; 27: 1196–1205. 38795956 10.1016/j.jval.2024.05.006 PMC11343728

[33] Babitsch B Gohl D von Lengerke T . Re-revisiting andersen's behavioral model of health services use: a systematic review of studies from 1998-2011. Psychosoc Med 2012; 9: Doc11. 10.3205/psm000089 PMC3488807 23133505

[34] Hajek A Kretzler B Konig HH . Determinants of healthcare use based on the Andersen Model: a systematic review of longitudinal studies. Healthcare (Basel) 2021; 9: 1354. 34683034 10.3390/healthcare9101354 PMC8544403

[35] Andersen RM . Revisiting the behavioral model and access to medical care: does it matter? J Health Soc Behav 1995; 36: 1–10. 7738325

[36] Luppa M Luck T Weyerer S , et al. Prediction of institutionalization in the elderly. A systematic review. Age Ageing 2010; 39: 31–38. 19934075 10.1093/ageing/afp202

[37] Wimo A Handels R Winblad B , et al. Quantifying and describing the natural history and costs of Alzheimer's disease and effects of hypothetical interventions. J Alzheimers Dis 2020; 75: 891–902. 32390617 10.3233/JAD-191055 PMC7369101

[38] Nguyen K-H TA C Green C . Where are we at with model-based economic evaluations of interventions for dementia? A systematic review and quality assessment. Int Psychogeriatr 2018; 30: 1593–1605. 30475198 10.1017/S1041610218001291

[39] Brennan A Chick SE Davies R . A taxonomy of model structures for economic evaluation of health technologies. Health Econ 2006; 15: 1295–1310. 16941543 10.1002/hec.1148

[40] de Vugt ME Verhey FR . The impact of early dementia diagnosis and intervention on informal caregivers. Prog Neurobiol 2013; 110: 54–62. 23689068 10.1016/j.pneurobio.2013.04.005

[41] Belger M Haro JM Reed C , et al. Determinants of time to institutionalisation and related healthcare and societal costs in a community-based cohort of patients with Alzheimer's disease dementia. Eur J Health Econ 2019; 20: 343–355. 30178148 10.1007/s10198-018-1001-3 PMC6438944

[42] Olsen C Pedersen I Bergland A , et al. Differences in quality of life in home-dwelling persons and nursing home residents with dementia - a cross-sectional study. BMC Geriatr 2016; 16: 137. 27400744 10.1186/s12877-016-0312-4 PMC4939817

[43] Jonsson L Tate A Frisell O , et al. The costs of dementia in Europe: an updated review and meta-analysis. Pharmacoeconomics 2023; 41: 59–75. 36376775 10.1007/s40273-022-01212-z PMC9813179

[44] Coley N Gallini A Garès V , et al. A longitudinal study of transitions between informal and formal care in Alzheimer disease using multistate models in the European ICTUS cohort. J Am Med Dir Assoc 2015; 16: 1104.e1–7. 10.1016/j.jamda.2015.09.010 26593306

[45] Janssen O Vos SJB Handels R , et al. Duration of care trajectories in persons with dementia differs according to demographic and clinical characteristics. J Am Med Dir Assoc 2020; 21: 1102–1107.e6. 32113914 10.1016/j.jamda.2020.01.008

[46] Joling KJ Janssen O Francke AL , et al. Time from diagnosis to institutionalization and death in people with dementia. Alzheimers Dement 2020; 16: 662–671. 32072728 10.1002/alz.12063 PMC7984226

[47] Handels R Herring WL Grimm S , et al. New IPECAD open-source model framework for the health technology assessment of early Alzheimer's disease treatment: development and use cases. Value Health 2025; 28: 511–518. 39094686 10.1016/j.jval.2024.07.009

[48] Yu CR Sommerlad A Sakure L , et al. Socially assistive robots for people with dementia: systematic review and meta-analysis of feasibility, acceptability and the effect on cognition, neuropsychiatric symptoms and quality of life. Ageing Res Rev 2022; 78: 101633. 35462001 10.1016/j.arr.2022.101633

[49] Vandepitte S Putman K Van Den Noortgate N , et al. Cost-effectiveness of an in-home respite care program to support informal caregivers of persons with dementia: a model-based analysis. Int J Geriatr Psychiatry 2020; 35: 601–609. 32011773 10.1002/gps.5276

[50] Martikainen J Valtonen H Pirttilä T . Potential cost-effectiveness of a family-based program in mild Alzheimer's disease patients. Eur J Health Econ 2004; 5: 136–142. 15452750 10.1007/s10198-003-0214-1

[51] Howard R Gathercole R Bradley R , et al. The effectiveness and cost-effectiveness of assistive technology and telecare for independent living in dementia: a randomised controlled trial. Age Ageing 2021; 50: 882–890. 33492349 10.1093/ageing/afaa284 PMC8099012

[52] Toot S Swinson T Devine M , et al. Causes of nursing home placement for older people with dementia: a systematic review and meta-analysis. Int Psychogeriatr 2017; 29: 195–208. 27806743 10.1017/S1041610216001654

[53] Tampi RR Bhattacharya G Marpuri P . Managing behavioral and psychological symptoms of dementia (BPSD) in the era of boxed warnings. Curr Psychiatry Rep 2022; 24: 431–440. 35781675 10.1007/s11920-022-01347-y

[54] *Surrogate endpoints in cost-effectiveness analysis for use in health technology assessment*. National Institute for Health and Care Excellence. https://www.cda-amc.ca/sites/default/files/MG%20Methods/surrogate-endpoints-report.pdf (2024). 10.1017/S0266462326103791 PMC13468940 42563624

[55] Gaugler JE Yu F Krichbaum K , et al. Predictors of nursing home admission for persons with dementia. Med Care 2009; 47: 191–198. 19169120 10.1097/MLR.0b013e31818457ce

[56] Vugt MED Stevens F Aalten P , et al. A prospective study of the effects of behavioral symptoms on the institutionalization of patients with dementia. Int Psychogeriatr 2005; 17: 577–589. 16185379 10.1017/S1041610205002292

[57] Aguero-Torres H von Strauss E Viitanen M , et al. Institutionalization in the elderly: the role of chronic diseases and dementia. Cross-sectional and longitudinal data from a population-based study. J Clin Epidemiol 2001; 54: 795–801. 11470388 10.1016/s0895-4356(00)00371-1

[58] Cepoiu-Martin M Tam-Tham H Patten S , et al. Predictors of long-term care placement in persons with dementia: a systematic review and meta-analysis. Int J Geriatr Psychiatry 2016; 31: 1151–1171. 27045271 10.1002/gps.4449

[59] Kerpershoek L de Vugt M Wolfs C , et al. Is there equity in initial access to formal dementia care in Europe? The Andersen Model applied to the Actifcare cohort. Int J Geriatr Psychiatry 2020; 35: 45–52. 31647572 10.1002/gps.5213 PMC6916585

[60] Kraijo H van Exel J Brouwer W . The perseverance time of informal carers for people with dementia: results of a two-year longitudinal follow-up study. BMC Nurs 2015; 14: 56. 26549986 10.1186/s12912-015-0107-5 PMC4636746

[61] Richters A Melis RJ van Exel NJ , et al. Perseverance time of informal caregivers for people with dementia: construct validity, responsiveness and predictive validity. Alzheimers Res Ther 2017; 9: 26. 28372581 10.1186/s13195-017-0251-0 PMC5379582

[62] Appendix J: Health economics. In Dementia: Assessment, management and support for people living with dementia and their carers. London: NICE, 2018. 30011160

[63] Rive B Grishchenko M Guilhaume-Goulant C , et al. Cost effectiveness of memantine in Alzheimer's disease in the UK. J Med Econ 2010; 13: 371–380. 20504112 10.3111/13696998.2010.491347

[64] Jutkowitz E Pizzi LT Shewmaker P , et al. Cost effectiveness of non-drug interventions that reduce nursing home admissions for people living with dementia. Alzheimers Dement 2023; 19: 3867–3893. 37021724 10.1002/alz.12964 PMC10524701

[65] Neumann PJ Hermann RC Kuntz KM , et al. Cost-effectiveness of donepezil in the treatment of mild or moderate Alzheimer's disease. Neurology 1999; 52: 1138–1145. 10214734 10.1212/wnl.52.6.1138

[66] Kansal AR Tafazzoli A Ishak KJ , et al. Alzheimer's disease Archimedes condition-event simulator: development and validation. Alzheimers Dement (N Y) 2018; 4: 76–88. 29687076 10.1016/j.trci.2018.01.001 PMC5910516

[67] Sculpher M Fenwick E Claxton K . Assessing quality in decision analytic cost-effectiveness models. PharmacoEconomics 2000; 17: 461–477. 10977388 10.2165/00019053-200017050-00005

[68] Green C Shearer J Ritchie CW , et al. Model-based economic evaluation in Alzheimer's disease: a review of the methods available to model Alzheimer's disease progression. Value Health 2011; 14: 621–630. 21839398 10.1016/j.jval.2010.12.008

[69] Boverhof BJ Redekop WK Visser JJ , et al. Broadening the HTA of medical AI: a review of the literature to inform a tailored approach. Health Policy Technol 2024; 13: 100868.

[70] Spijker A Vernooij-Dassen M Vasse E , et al. Effectiveness of nonpharmacological interventions in delaying the institutionalization of patients with dementia: a meta-analysis. J Am Geriatr Soc 2008; 56: 1116–1128. 18410323 10.1111/j.1532-5415.2008.01705.x

[71] Ettema TP Droes RM de Lange J , et al. The concept of quality of life in dementia in the different stages of the disease. Int Psychogeriatr 2005; 17: 353–370. 16252370 10.1017/s1041610205002073

[72] Gaugler JE Wall MM Kane RL , et al. The effects of incident and persistent behavioral problems on change in caregiver burden and nursing home admission of persons with dementia. Med Care 2010; 48: 875–883. 20733529 10.1097/MLR.0b013e3181ec557b

[73] Diaz I Lee H Kiciman E , et al. Sensitivity analysis for causality in observational studies for regulatory science. J Clin Transl Sci 2023; 7: e267. 10.1017/cts.2023.688 PMC10877517 38380390

[74] Neumann PJ Garrison LP Willke RJ . The history and future of the “ISPOR Value Flower": addressing limitations of conventional cost-effectiveness analysis. Value Health 2022; 25: 558–565. 35279370 10.1016/j.jval.2022.01.010

[75] Cookson R Mirelman AJ Griffin S , et al. Using cost-effectiveness analysis to address health equity concerns. Value Health 2017; 20: 206–212. 28237196 10.1016/j.jval.2016.11.027 PMC5340318

[76] Hoedemakers M Tsiachristas A Rutten-van Mölken M . Moving beyond quality-adjusted life-years in elderly care: how can multicriteria decision analysis complement cost-effectiveness analysis in local-level decision making. Value Health 2022; 25: 1717–1725. 35623974 10.1016/j.jval.2022.04.1728

[77] IJzerman MJ Koffijberg H Fenwick E , et al. Emerging use of early health technology assessment in medical product development: a scoping review of the literature. Pharmacoeconomics 2017; 35: 727–740. 28432642 10.1007/s40273-017-0509-1 PMC5488152

