# Supplementary material for: Assessing technologies in dementia care: A conceptual health-economic model
Source: J Alzheimers Dis. 2026 Jan 28;110(1):471–83. doi: 10.1177/13872877251415203 (PMC12960762; doi:10.1177/13872877251415203)
Supplement: sj-docx-1-alz-10.1177_13872877251415203 - Supplemental material for Assessing technologies in dementia care: A conceptual health-economic model [file sj-docx-1-alz-10.1177_13872877251415203.docx]

## **Supplemental Material**

## **Assessing technologies in dementia care: A conceptual health-economic model**

## **Supplemental Material 1. Expert Discussions**

### ***Purpose***

The expert discussions and workshop aimed to specify the technology’s intended purpose and target population, the proposed impact pathway of using the technology, and the expected health, cost, and resource impacts compared to current practice. Insights from these discussions informed the conceptual structure and assumptions of the health-economic model.

Experts were invited through the QoLEAD consortium network based on their ongoing involvement in dementia care, technology development, care organization, and health-economic modelling. They were not purposively selected but represented complementary perspectives across these domains.

### ***Format and organization***

In total, **ten sessions** were held between [September 2023 – November 2024]:

- **Nine unstructured group discussions**, each lasting 1 hours, involving 18 experts with backgrounds in e-health, AI, industrial design, cognitive neuropsychology, virtual agents and social robots, dementia care and health economics in the Netherlands.
- **One half-day workshop** organized with predefined discussion topics and structured team activities.

All sessions were facilitated by the first, second, and/or last authors (JF, RH, EB), who contributed methodological and health-economic expertise but were **not included as participants** in the expert list. Additionally, **seven PhD students** from the QoLEAD consortium attended one or more sessions, contributing insights from their respective research domains; however, they were not listed as experts (see Supplemental Table 1).

### ***Discussion focus***

Although the nine group discussions were unstructured, conversations focused on three recurring themes shaped by the participants’ expertise:

- **Technology characterization:** describing the function, target group, design approach, and early evidence of potential impact from pilot programs.
- **Impact pathway:** defining the role of AI in current dementia care trajectory and identifying relevant care states and transitions along the care trajectory.
- **Data needs:** discussing the availability, limitations, and gaps in existing data for model parameterization and validation.

### ***Workshop description***

The final **half-day workshop** adopted a structured format and aimed to synthesize and validate insights from previous discussions.

Its objectives were to:

- Define the **impact pathways** through which AI-based technologies may influence quality of life and resource use;
- Identify potential **surrogate or intermediate outcomes** for measuring short-term impact in pilot studies; and
- Explore the **association of surrogate outcome and long-term outcome**.

Participants worked in four interdisciplinary teams, each mapping the impact pathway for a selected technology (a cooking agent, a music robot, and a tool for social enrichment). In the second part of the workshop, discussions focused on how to quantify impact, identify measurable surrogate outcomes, and recognize common challenges in evaluating non-pharmacological interventions within short-term pilots.

### ***Documentation and use***

Findings from both the expert discussions and the workshop were synthesized and confirmed in the technology’s **impact pathway** and model structure (see Figures 1 and 2 and Supplemental Figures 1 and 2). A summary of participant expertise and affiliations is provided in Supplemental **Table 1**.

**Supplemental Table 1.** Overview of experts participating in the model conceptualization discussions.

| Number | Expertise/Role | Affiliation | Sessions attended | Workshop attend |
| --- | --- | --- | --- | --- |
| 1 | Assistant professor (Virtual agents, social robots and artificial intelligence) | Radboud University | 3 | Yes |
| 2 | Professor (Economic evaluation and health technology assessment) | RIVM/ University of Groningen | 2 | / |
| 3 | Professor (gero-psychologist, long-term care) | Radboud University Medical Center | 2 | / |
| 4 | Professor (Industrial design) | Tilburg University | 2 | / |
| 5 | Professor (long-term care, Gerontechnology) | Eindhoven University of Technology/Vilans | 1 | / |
| 6 | Senior researcher (palliative care for people living with dementia, clinical and neuropsychology) | Tilburg University | 3 | Yes |
| 7 | Professor (artificial intelligence, cognitive neuropsychology, human-technology interaction) | Eindhoven University of Technology | 3 | Yes |
| 8 | Professor (Artificial Intelligence, communication science) | Radboud University | 2 | Yes |
| 9 | Professor (Diagnosis and treatment of early dementia, young-onset dementia, E-health support for informal caregivers) | Maastricht University | 1 | / |
| 10 | Associate professor (Clinical Neuropsychology, music psychology and cognitive neuroscience) | Leiden University | / | Yes |
| 11 | Technology developer 1 | AUMENS | 1 | / |
| 12 | Technology developer 2 | AUMENS | 2 |  |
| 13 | Psychogerontologist | Alzheimer Nederland | / | Yes |
| 14 | Policy officer | TanteLouise | / | Yes |
| 15 | Project manager | TIGNL BV, Technology Investment Group / JAIN Foundation, | / | Yes |
| 16 | Assistant professor (long-term care, aging-in-place) | Maastricht University | 1 | / |
| 17 | Professor (social and demographic change, life course analysis,) | University Medical Centre Groningen | 1 | / |
| 18 | Researcher (eHealth, Dementia, Health Psychology and Implementation Science) | Maastricht University | 1 | / |
| Other attendees | | | | |
|  | PhD student (technology development) | Radboud University | 1 | Yes |
|  | PhD student (technology development) | Delft University | 1 | Yes |
|  | PhD student (AI-tool, quality of life) | Maastricht University | 1 | Yes |
|  | PhD student (human-technology interaction) | Eindhoven University of Technology | 2 | Yes |
|  | PhD student (co-creation and participatory design with people with dementia and AI) | Eindhoven University of Technology | / | Yes |
|  | PhD student (responsible innovation) | Utrecht University | 1 | / |
|  | PhD student (psychology, technology) | Radboudumc | 2 | Yes |

*Description of technologies in QoLEAD and impact ways to health economic outcomes*

**Precision Care Tool** aims to predict risk factors of quality of life in people with dementia by analyzing clinical records from nursing homes. It then alerts the caregivers and allows them to prevent or manage the crisis.

*Impact pathway:* By providing early warning signals for behavioral symptoms or other risk factors related to quality of life, the technology may improve the coping strategy, potentially leading to improved quality of life for persons with dementia and reduced caregiver stress from care uncertainties. This, in turn, could make community-based care more sustainable, reduce the need for specialized care, thereby reducing healthcare costs associated with care use.

**Supplemental Figure 1.** The impact pathway of a precision care tool.


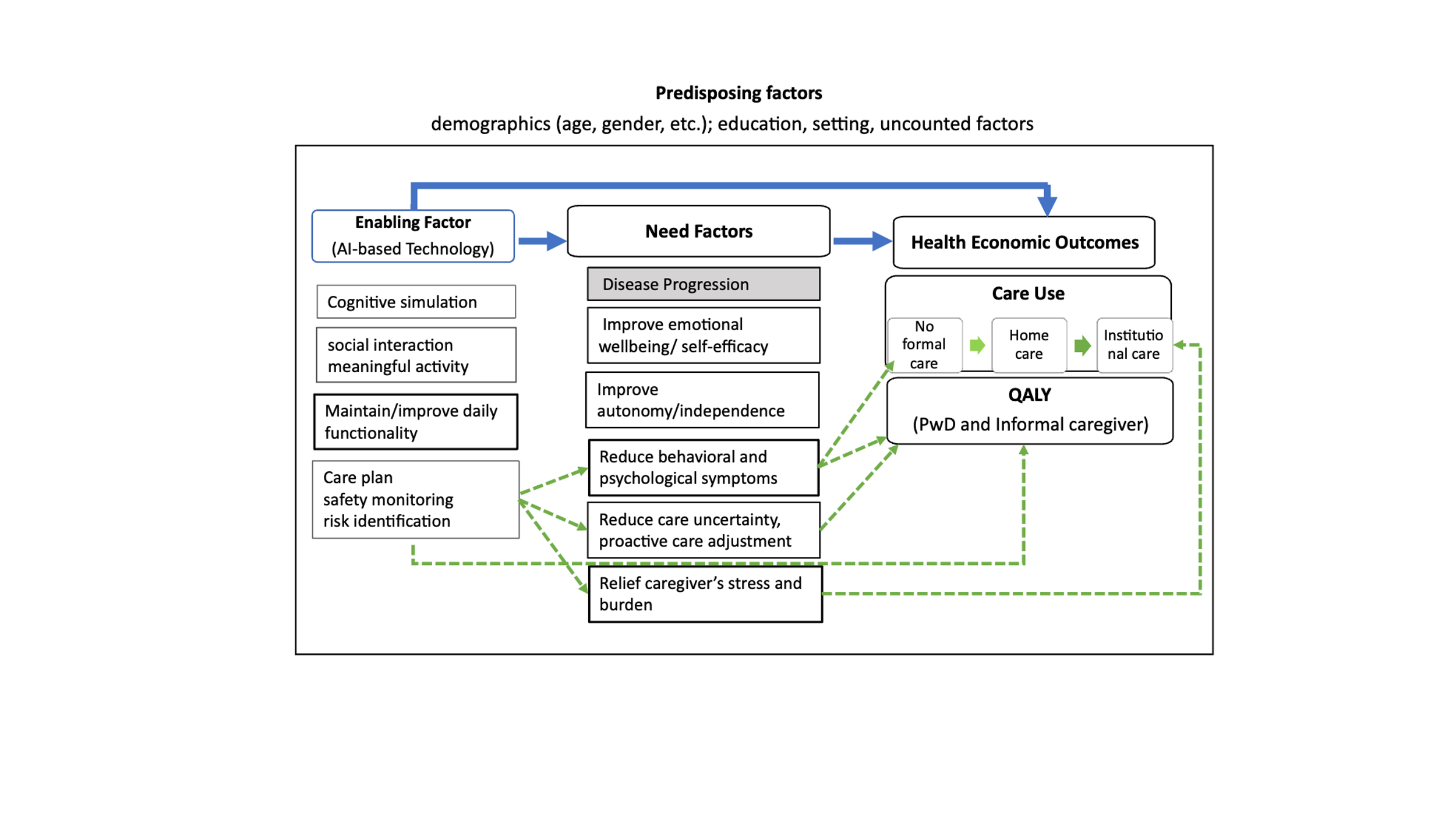


**Musical Social Robot** is designed to engage persons with dementia through music and social interaction, guiding and enriching their daily activities. It provides personalized musical experiences with cognitive reminiscence events, tailored to the individual’s preferences and cognitive abilities. The robot may also facilitate social interactions by encouraging participation in group activities, reminiscing through music, and fostering connections with caregivers and peers.

*Impact pathway:* Vy enhancing social engagement, stimulating cognitive functioning and enriching meaningful activities, it can help improve the overall quality of life for individuals with dementia. Increased social interaction may contribute to enhanced emotional well-being, reduced feelings of isolation, and improved mood. Additionally, by reducing stress and agitation, the robot may alleviate caregiver burden. This reduction in caregiver strain could lead to a postponement of formal caregiving services or institutionalization, ultimately resulting in cost savings in dementia care.

**Supplemental Figure 2.** The impact pathway of a music social robot.


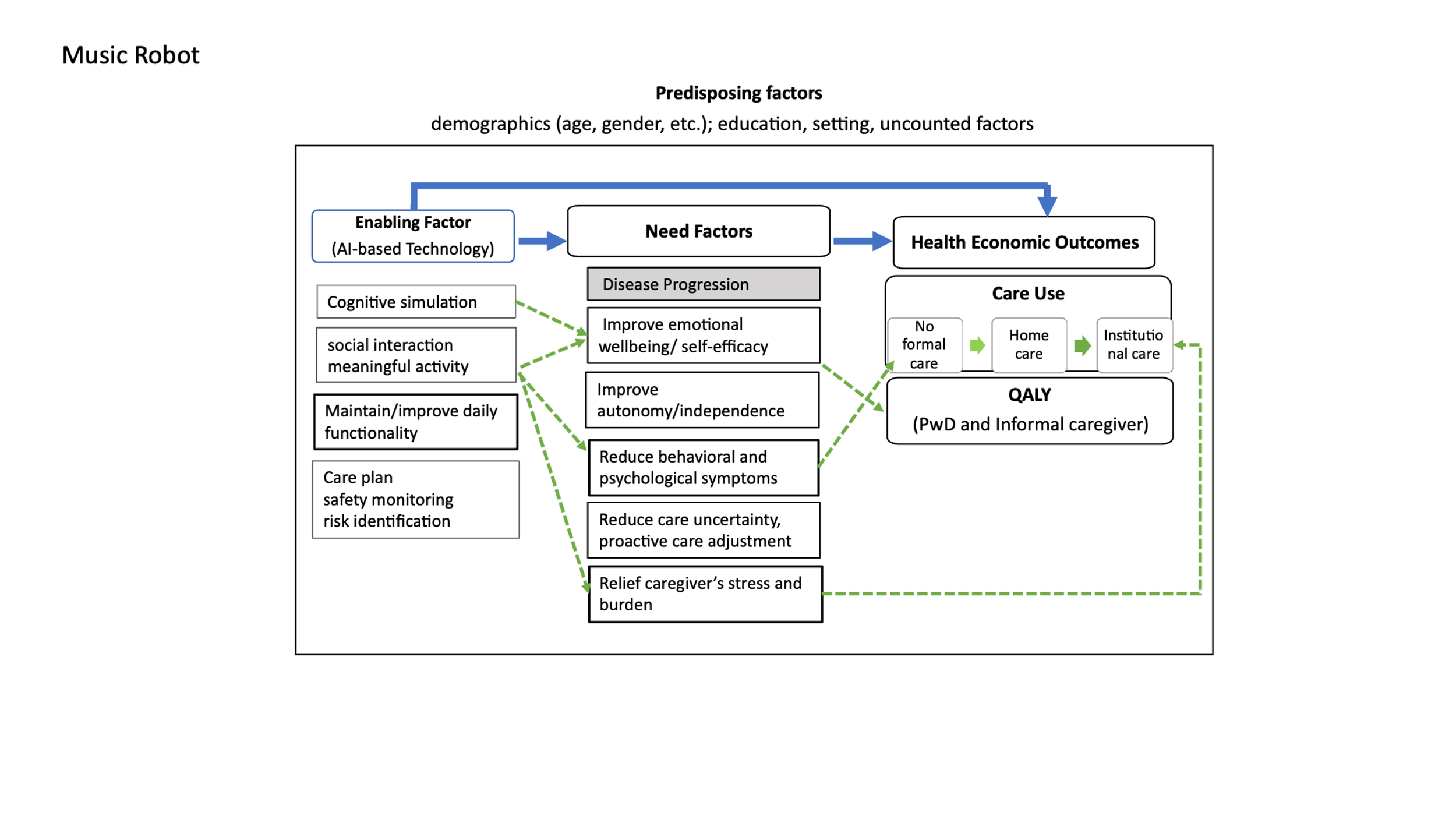


## **Supplemental Material 2**

**Supplemental Table 2.** Expected outcomes in the pilot and their long-term health economic implications.

| **Category** | **Expected impact of the technology listed by experts - potential surrogate outcomes** | **Predict Final outcome** |
| --- | --- | --- |
| Activities of daily living | Ability to perform daily activity | Care setting transition |
| Cognition | Cognitive simulation (activation) | Quality of Life |
| Behavioral and psychological symptoms | Wandering  Agitation  Depression | Care setting transition  Or Quality of Life |
| Feeling valued and respected by others.  A sense of who you are | Self-worth/meaningfulness  Self-respect  Dignity  Stigma  Outside image  Autonomy  Self-efficacy  Independency  Self-esteem | Quality of Life |
| Emotional wellbeing | Loneliness  Mood  Stress | Quality of Life |
| Communication  Importance of relationship  Having a laugh | Engagement in activities  Social health  Social network quality and quantity  Social-connection/relations/dynamic | Quality of Life |
| Feeling safe and secure | Safety  Quality of care | Quality of Life |
| Care Use | Caregiving hours  Frequency in contact with formal caregiver  Reduced crisis  Adverse events | Care Use |
| Caregiver burden | Caregiver stress, satisfaction, relief | Care Transition  Caregiver- quality of life |
| Technology Metrics * | Quality of system  Frequency of use  Ease of use  Learning curve  Spillover effects  …. |  |

*Not relevant to health economic outcomes

## **Supplemental Material 3**

Care settings were defined following current Dutch care use pattern^1^: no formal care (care support from family and unpaid carer, GP care only), home care (receiving any formal form of care support for ≥30 days per year, including domestic assistance, personal care, nursing support, or daycare), and institutional care (nursing home, residential home, or long-stay psycho-geriatric care for ≥30 days per year). The resource use and cost in each care state were defined as below:

- **No formal care:** includes informal care costs and medical care costs (hospital admissions, emergency visits, general practitioner consultations).
- **Home care:** This state reflects the initiation of formal home care. It adds formal home care costs (including home nursing, domestic help, and day care services) in addition to informal care costs and medical care costs.
- **Institutional care:** This state reflects admission to nursing home. It includes a fixed institutional care cost component and average costs related to informal care and medical care of people in an institutional setting.

**Reference**

1. Janssen O, Vos SJB, Handels R, et al. Duration of care trajectories in persons with dementia differs according to demographic and clinical characteristics. *J Am Med Dir Assoc* 2020; 21: 1102-1107.e6.
